# Retraction Note: Single-atoms (N, P, S) encapsulation of Ni-doped graphene/PEDOT hybrid materials as sensors for H_2_S gas applications: intuition from computational study

**DOI:** 10.1038/s41598-024-70290-0

**Published:** 2024-08-21

**Authors:** Innocent Benjamin, Hitler Louis, Festus O. Ogungbemiro, Daniel C. Agurokpon, Bassey O. Ekpong, Terkumbur E. Gber, Anthony M. S. Pembere

**Affiliations:** 1 Computational and Bio‑Simulation Research Group, University of Calabar, Calabar, Nigeria; 2 Department of Microbiology, University of Calabar, Calabar, Nigeria; 3 Department of Pure and Applied Chemistry, University of Calabar, Calabar, Nigeria; 4 Department of Chemistry, Federal University of Lafia, Lafia, Nassarawa State, Nigeria; 5 Department of Chemistry, Jaramogi Odinga University of Science and Technology, Bondo, Kenya

Retraction of: *Scientific Reports* 10.1038/s41598-023-46153-5, published online 01 November 2023

The Editors have retracted this Article.

After the publication of the Article, concerns were raised about incomplete descriptions of the methods for the molecular dynamics simulation and seemingly unrealistic and uncontrolled temperatures for the simulations shown in Figure 8. The Editors requested raw data; however, the information provided by the authors was not satisfactory. Post-publication peer review by experts identified additional concerns regarding the short time window used for the molecular dynamics simulation and the oversimplification of the quantum mechanical approximations that have been employed, which introduce significant uncertainty about the results presented.

The Editors have therefore lost confidence in the results and conclusions presented in this Article.

Innocent Benjamin, Festus O. Ogungbemiro, Daniel C. Agurokpon, Bassey O. Ekpong, Terkumbur E. Gber, and Anthony M. S. Pembere have not responded to correspondence from the Editors about this retraction. Hitler Louis did not state explicitly whether they agreed or disagreed with this retraction.

